# Serum metabonomics of acute leukemia using nuclear magnetic resonance spectroscopy

**DOI:** 10.1038/srep30693

**Published:** 2016-08-02

**Authors:** Syed Ghulam Musharraf, Amna Jabbar Siddiqui, Tahir Shamsi, M. Iqbal Choudhary, Atta-ur Rahman

**Affiliations:** 1 Dr. Panjwani Center for Molecular Medicine and Drug Research, International Center for Chemical and Biological Sciences, University of Karachi, Karachi-75270, Pakistan; 2 H.E.J. Research Institute of Chemistry, International Center for Chemical and Biological Sciences, University of Karachi, Karachi-75270, Pakistan; 3 National Institute of Blood Diseases & Bone Marrow Transplantation, Karachi-75300, Pakistan; 4 Department of Chemistry, College of Science, King Saud University, Riyadh-1145, Saudi Arabia

## Abstract

Acute leukemia is a critical neoplasm of white blood cells. In order to differentiate between the metabolic alterations associated with two subtypes of acute leukemia, acute lymphoblastic leukemia (ALL) and acute myeloid leukemia (AML), we investigated the serum of ALL and AML patients and compared with two controls (healthy and aplastic anemia) using ^1^H NMR (nuclear magnetic resonance) spectroscopy. Thirty-seven putative metabolites were identified using Carr-Purcell-Meiboom-Gill (CPMG) sequence. The use of PLS-DA and OPLS-DA models gave results with 84.38% and 90.63% classification rate, respectively. The metabolites responsible for classification are mainly lipids, lactate and glucose. Compared with controls, ALL and AML patients showed serum metabonomic differences involving aberrant metabolism pathways including glycolysis, TCA cycle, lipoprotein changes, choline and fatty acid metabolisms.

Leukemia is among the major causes of mortality around the world, especially in children^1^. The main etiology of acute leukemia is the malignant alteration of lymphoid or myeloid cells into primitive and undifferentiated cells. About half of the leukemia cases are associated with acute leukemia. Under its sub-category, acute lymphoblastic leukemia (ALL) is being the most widespread cancer in children while over 80% of acute myeloid leukemia (AML) cases that occur in adults. The 5-year survival rate for leukemia is 54%. However, 5-year relative survival rates for children under age 15 is 89% for ALL and 60% for AML^2^. According to the report from Globocan 2012, approximately 0.35 million new cases were reported for leukemia, and 0.26 million children and adults died with leukemia^3^.

Blood and bone marrow tests are important both for precise diagnosis, while immunological and cytogenetic studies are helpful for prognostic information. Blood tests usually do not unambiguously establish the presence of leukemia during the early stages of the disease or remission. Morphological examination is generally used for primary diagnosis of AL, and differentiation between ALL to AML^4^. For diagnosis of leukemia, examination of smears of bone marrow aspirates, immuno-histochemical, and immunologic methods are used^5^. Despite the use of these methods, many people are not diagnosed early enough because often the symptoms are vague and unspecific. Without timely treatment patients with acute leukemia do not survive more than a few weeks^6^. Therefore, it is crucial to identify early detectable non-invasive biomarkers for leukemia, to save the lives of millions of patients through timely interventions.

Metabolomics is progressively being used as a biomarker discovery tool. Tissues and biofluids both have been used for the discovery of early diagnostic metabolite biomarkers of cancer in recent years^7^,^8^. However, biofluids are increasingly used to study metabolic variations. Nuclear magnetic resonance (NMR) is now been used as a robust tool in the field of metabolomics. It offers unbiased information about metabolite profiles. A holistic view of the metabolites can be obtained by NMR-based metabolomics in many circumstances, and therefore it is found to be specially suited for metabolomic studies of biofluids^9^. Researchers have reported few recent studies on leukemia. For instance, tumor microenvironment for childhood ALL has been done through high-resolution magnetic resonance spectrometry (MRS) and gas chromatography mass spectrometry (GC-MS) based on fatty acids analysis^10^. Another pediatric ALL biomarker study was carried out using ultra pressure liquid chromatography^11^. A metabonomic study for de novo AML was also done by using ^1^H NMR spectroscopy^12^. However, no study using other control diseases has been carried out; and only one control of healthy individuals was included in all reported studies.

In this study, serum metabolite profiling of two sub-classes of acute leukemia (ALL and AML) was carried out with two categories of controls, aplastic anemia (APA) and healthy individuals, using ^1^H-NMR. Our objective was to identify differentiative metabolites between the two classes of acute leukemia and to identify key metabolites that can be used for disease diagnosis.

## Methods

### Sample collection

The study was approved by the Independent Ethics Committee, International Center for Chemical and Biological Sciences, University of Karachi and by the collaborating hospital. Written informed consent was obtained from all patients and healthy individuals prior to collection of data and samples. All the sample preparation methods were carried out in accordance with the approved guidelines. 96 patients were enrolled for this study with ALL, AML and APA (32 for each disease) from National Institute of Blood Diseases & Bone Marrow Transplantation, Karachi, Pakistan. These patients were diagnosed for the first time and had not received any prior treatment. The patients with leukemia secondary to other malignancies or transformed from myelodysplastic syndrome were not included in this study. To avoid the age and gender effects, 32 age- and gender-matched healthy control samples were also recruited. Sample details are mentioned in the Supplementary Table S1.

Serum samples were collected from the participants after overnight fasting (for at least 10 h) in BD vacutainer tubes (BD, Franklin Lakes, NJ, USA, REF 367381), coated interior with silicone as clot activator. The serum was separated after half an hour by centrifugation at 4,500 rpm for 10 min at 4 °C. Finally, the serum was aliquoted, and frozen at −80 °C till sample preparation.

### Sample preparation and analysis

The serum was thawed on ice just before the analysis and vortexed. 200 μL of the homogenized serum was mixed with 400 μL of D_2_O and centrifuged for 5 min at 6,000 rpm. 550 μL of this mixture was transferred to 5 mm NMR tube.

The ^1^H NMR spectra were acquired using a 500-MHz Bruker Avance AV-500 spectrometer (Bruker Biospin, Rheinstetten, Germany), equipped with a z-gradient probe at 300.0 K. Three 1D experiments were acquired for every sample. The first experiment was the standard ^1^H NMR spectrum (NOESY) with water suppression. The spectrum was acquired using a 90° flip angle with a recycle delay of 4 seconds, 10-kHz spectral width, 32,768 data points, 362 receiver gain, 0.1 second mixing time, total 128 scans, and four dummy scans. The second experiment was the diffusion edited ^1^H NMR spectrum with water suppression to select signals of large molecules. The spectrum was acquired with a recycle delay of 4 seconds, 10-kHz spectral width, 32,768 data points, 574.7 receiver gain, 0.1 second diffusion delay, 1 ms pulse field gradient, total 128 scans and four dummy scans. The third experiment wais the T_2_ edited ^1^H NMR spectrum (Car-Purcell-Meiboom-Gill (CPMG)) with water suppression in order to have enhanced visualization of low molecular weight compounds. The spectrum was acquired with a recycle delay of 4 seconds, 10-kHz spectral width, 32,768 data points, 362 receiver gain, 30 ms total spin-echo time, total 256 scans, and 16 dummy scans. The acquired NMR spectra were manually corrected for the phase and the baseline with TopSpin 2.1 (Bruker Biospin, Germany). All chemical shifts were manually referenced to the anomeric proton signal of glucose at 5.23 ppm.

In addition to these experiments, few 2D experiments were also performed on selected samples for peak assignments to molecules using existing databases^13^ and previously reported literature^14^,^15^. It includes ^1^H-^1^H correlation spectroscopy (COSY), ^1^H-^1^H *J*-resolved, and ^1^H-^13^C hetero nuclear single-quantum correlation (HSQC) spectroscopy.

### Chemometric analysis

The spectral region *δ* 13 to −2 of every spectra (total 384 spectra) was equally divided into rectangular buckets of 0.04 ppm using AMIX version 3.9 (Bruker Biospin, Rheinstetten, Germany), excluding the region *δ*4.90 to 4.66 in order to eliminate residual water saturation signals. Further, these bucket tables were exported to SIMCA-P package (version 14, Umetrics, Umeå, Sweden). All the data was normalized to unit area (to avoid concentration differences among metabolites), scaled using pareto scaling algorithm (to give equal weight to all variables despite their absolute value) and auto fitted for multivariate analysis.

Principal component analysis (PCA) was done first to create an overview, and to remove outliers. It is a statistical procedure that uses an orthogonal transformation to convert a set of observations of possibly correlated variables into a set of values of linearly uncorrelated variables called principal components. The number of principal components is less than or equal to the number of original variables. Partial least squares discriminant analysis (PLS-DA) and orthogonal partial least squares discriminant analysis (OPLS-DA) were then performed, which were used to decrease the potential role of intergroup inconsistency and to further amplify differences among the samples. PLS is a versatile algorithm, which can be used to predict variables. Classification with PLS is termed PLS-DA. This algorithm has many favorable properties for dealing with multivariate data; the most important of which is how variable collinearity is dealt with, and the model’s ability to rank variables’ predictive capacities within a multivariate context. OPLS-DA is an extension of PLS-DA which seeks to maximize the explained variance between groups in a single dimension or the first latent variable (LV), and separate the within group variance (orthogonal to classification goal) into orthogonal LVs. The variable loadings from a validated O-PLS-DA model can be used to rank all variables with respect to their performance for discriminating between groups. The default method of 7-fold internal cross validation of the software was applied, from which Q^2^Y (predictive ability parameter, estimated by cross-validation), and R^2^Y (goodness of fit parameter) values were extracted. Those parameters were used for the evaluation of the quality of the models obtained. CV-ANOVA and permutation methods were also employed on the models for their further validities. To identify the variations between groups of samples, the corresponding loading plots and the variable importance in projection (VIP) list of each model were carefully inspected, and finally used to identify which variables were important for discrimination between the groups.

## Results

**Figure 1:**
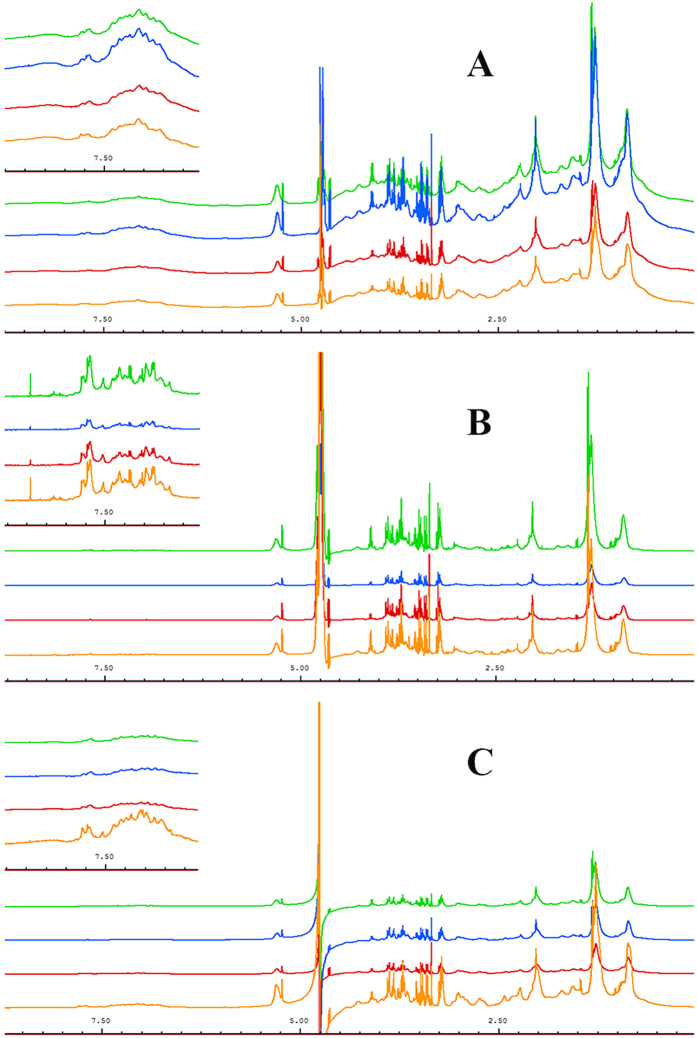
Stacked view of ^1^H NMR spectra of blood serum from ALL (green), AML (blue), APA (red) and healthy control (yellow). (**A**) standard 1D, (**B**) T_2_-edited (CPMG), (**C**) diffusion edited. The low field region (*δ* 6–9) is vertically projected 10 times relative to the rest of the spectrum.

**Figure 2:**
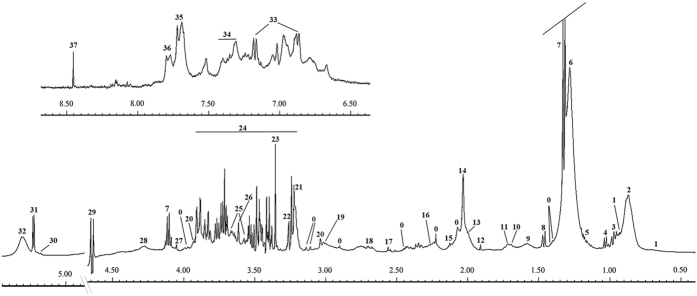
Assignments of the ^1^H-NMR signals of a representative 500 MHz 1D-CPMG ^1^H-NMR average spectrum of a healthy serum sample measured at 310 K. A, full spectrum (*δ* 5.50–0.5 ppm) and magnification of aromatic region (*δ* 9.00–6.50 ppm). Peak assignments: 0, unidentified; 1, cholesterol; 2, lipids (–CH_3_) (mainly LDL/VLDL); 3, leucine; 4, valine; 5, 3-hydroxybutyrate; 6, lipids (CH_2_)n (mainlyLDL/VLDL); 7, lactate; 8, alanine; 9, adipicacid; 10, arginine; 11, lysine; 12, acetate; 13, lipids (CH_2_–C=C); 14, acetyl signals from glycoproteins; 15, glutamine; 16, lipids (CH_2_–CO); 17, citrate; 18, lipids (CH=CH–CH_2_–CH=CH–); 19, Albumin lysyl; 20, creatine; 21, choline; 22, Trimethylamine N-oxide; 23, Proline; 24, glucose; 25, glycerol; 26, Myo-inositol; 27, creatinine; 28, threonine; 29, β-glucose; 30, glycerol of lipids; 31, α-glucose; 32, lipids (–CH=CH–); 33, tyrosine; 34, phenylalanine; 35, histidine; 36, 1-methylhistidine; 37, formate.

Thirty-two serum samples from each group (ALL, AML, APA, and healthy control) were included in this study. Three different 1D NMR experiments were performed against each sample. Figure 1 shows stacked view of average spectra of each group in which Fig. 1A is the standard ^1^H-NMR spectra, illustrating broader peaks of some proteins and their conjugates (*δ* 0–2.5). However, in the second experiment using the CPMG sequence, these broader signals were greatly reduced and ultimately gave clear signals of low molecular weight metabolites especially in low and higher field region (Fig. 1B). Similarly, diffusion edited spectra in Fig. 1C showed broader peak profile of macromolecules. Hence, the signals were assigned mainly based on average spectrum obtained using the CMPG sequence as in reported literature^14^,^15^ and online databases (Fig. 2). Major assignments were the typical metabolites present in the serum including glucose, sugars, amino acids, organic acids etc. Many resonances from fatty acyl chain of lipoproteins and glycoproteins were also seen in the CPMG spectrum. However, these were more broader in the diffusion edited and standard ^1^H spectra. Visual inspection of all these spectra showed a clear reduction in the concentration of almost all metabolites in AML and APA in comparison to healthy controls, while ALL showed close correlation with the healthy controls (Fig. 1B).

**Figure 3:**
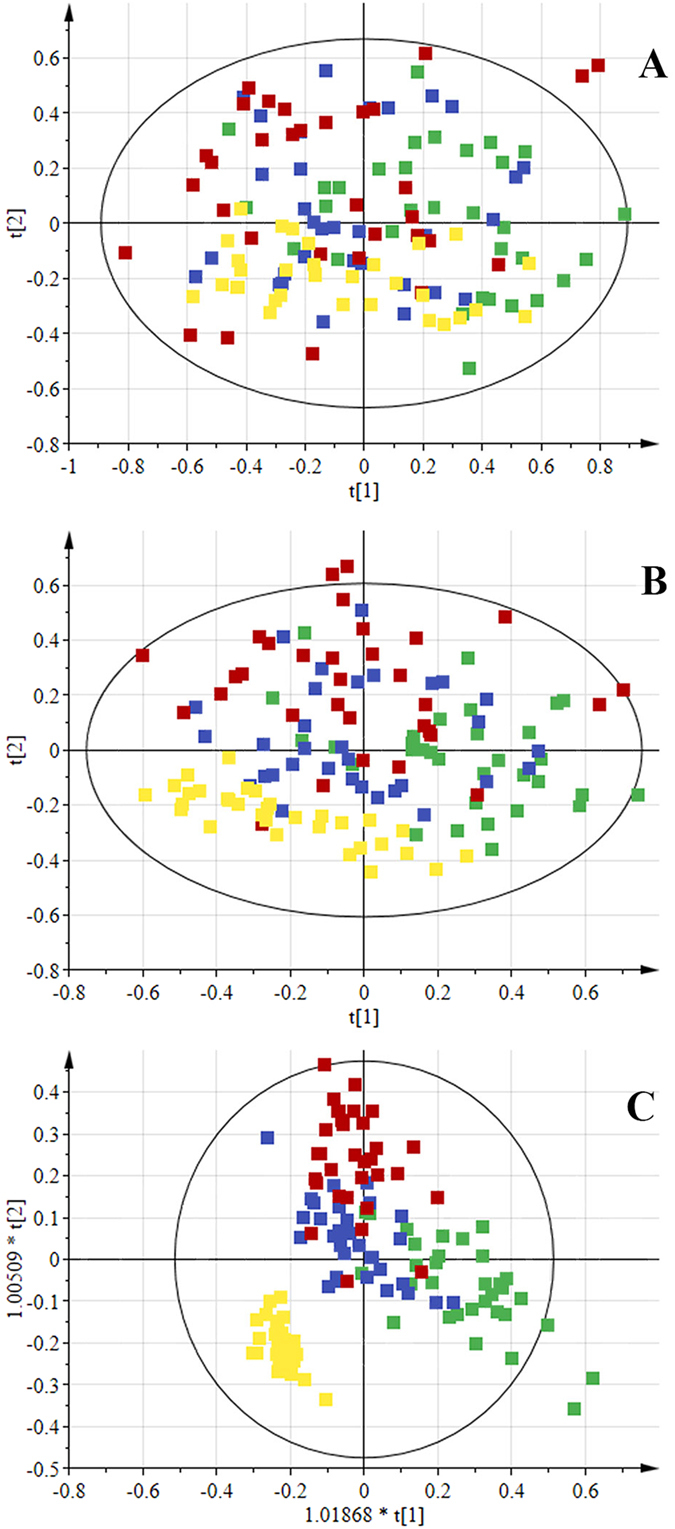
Scores scatter plots (**A**) PCA, (**B**) PLS-DA and (**C**) OPLS-DA of ^1^H CPMG NMR spectra of serum from ALL (green), AML (blue), APA (red) and healthy control (yellow).

**Figure 4:**
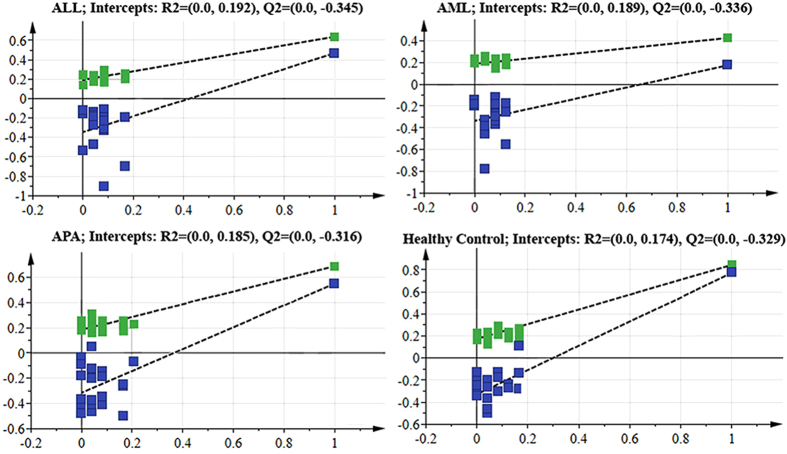
Permutation plots for the OPLS-DA model showing R2 (green) and Q2 (blue) values.

For the confirmation of these visual observations, multivariate analysis on the data was performed. The unsupervised PCA analysis was first performed in order to obtain a trend of separation of samples according to groups (Fig. 3A). No differences related to age and gender were found in PCA analysis, however five samples showed strong outlier behavior and lay outside the Hotelling’s 99% confidence limit (Supplementary Figure S1). Detailed review of these samples based on the clinical history of these patients revealed that either they were diabetic or hypertensive. Hence these samples were excluded from the study. For further separation of groups, PLS-DA and OPLS-DA models were also generated. Score plot in Fig. 3B showed slight separation of the healthy group from the others, while Fig. 3C showed clear separation among the groups. Table 1 shows the summarized results of these models. Overall, the model based on CPMG spectra gave the best results with 83.38% and 90.34% classification rate in PLS-DA and OPLS-DA models, respectively. Further validation of these models was also performed by using CV-ANOVA (Supplementary Table S2) and permutation tests (Fig. 4). The CV-ANOVA is based on cross validation for the estimation of independent predictors (OPLS-scores) and predictive residuals. The use of CV predictive residuals makes the CV-ANOVA more reliable than ordinary ANOVA. Lower *p*-value showed that the group separation is significant. Similarly, the Permutations Plot helps to assess the risk that the model is spurious, i.e., the model just fits the training set well but does not predict Y well for new observations. The idea of this validation is to compare the goodness of fit (R2 and Q2) of the original model with the goodness of fit of several models based on data where the order of the Y-observations has been randomly permuted, while the X-matrix has been kept intact. The plot in Fig. 4 strongly indicates that the original model is valid. The criteria for validity are: All blue Q2-values to the left are lower than the original points to the right. *Or* the blue regression line of the Q2-points intersects the vertical axis (on the left) at, or below zero. Receiver Operating Characteristics (ROC) curve was also produced for OPLS-DA, and area under the curve (AUC) were calculated (Supplementary Figure S2). The sensitivity is a measure of how well the model is able to classify correctly samples of the class of cases, while the specificity measures how well the model can predict samples from the class of controls. By plotting the sensitivity against 1-specificity for different values of the discrimination threshold, a ROC curve can be defined. The ROC curve provides a spectrum of performance assessments and the area under the *ROC (AUROC*) is commonly used as diagnostic statistics of models. The *AUROC* values range from 1 (perfect discrimination between classes) and 0 (0.5 and lower usually means no discrimination at all). The healthy control group showed 100% sensitivity and selectivity for the model while the values for AUC for disease groups were found to be 0.97, 0.92, and 0.96 for ALL, AML and APA, respectively.

**Figure 5:**
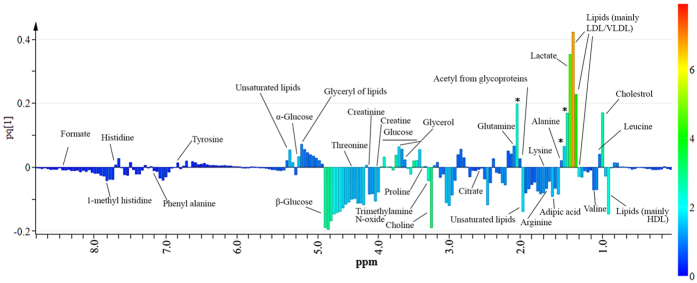
OPLS-DA loadings plot colored as a function of VIP. Assignment of main signals is indicated (unassigned signals with high VIP are marked with an asterisk).

The metabolites responsible for class discrimination were identified by performing color coding of the first component of OPLS-DA loadings on the basis of Variable Importance in Projection (VIP) values and VIP values > 1 were assigned to different compounds (Fig. 5). The color-coded loading plot showed that the major lipoprotein class appeared at different regions of spectra with different proportions. This could be due to the splitting of fatty-acyl chain resonances into positive and negative signals that correspond to LDL+ VLDL, and HDL lipoprotein, respectively^16^,^17^. This assignment was further validated by the negative signal at 3.21 ppm from choline, as it is already known that phosphatidylcholine is a major phospholipid^18^. With reference to the small metabolites, the OPLS-DA loadings with high VIP values (>1.0) were due to lactate, some amino acids (valine, alanine, lysine/arginine, glutamine, tyrosine, threonine and histidine), acetate, citrate, formate, adipic acid and glucose, signifying their importance when their concentrations were compared with disease and healthy control. Furthermore, a few other signals in the low frequency regions (indicated with asterisks in Fig. 4) showed high VIP values, and can be used for sample discrimination. However, at this stage their identification was not carried out.

**Figure 6:**
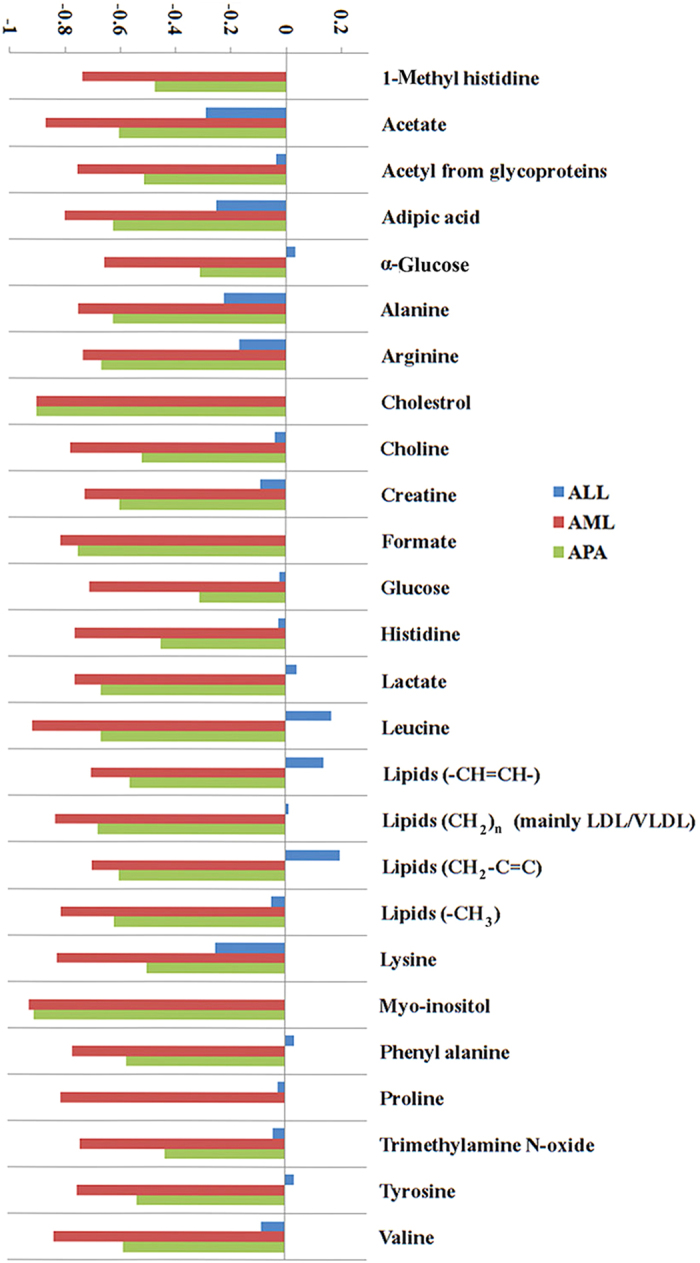
Average changes relative to healthy control of main metabolites contributing to the discrimination between serum of cancer patients and of healthy subjects.

To validate the differences accentuated by loadings examination, particular signals in the CPMG spectra have been integrated, and the mean areas between control and cancer groups have been compared. Figure 6 shows the certain discriminating metabolites and their relative variations in ALL, AML, and APA samples relative to the control healthy serum. Except for a few metabolites, the concentrations of all the remaining metabolites were decreased in the other three diseases.

## Discussion

Warburg suggested that disturbance in glycolysis is a cause of many cancers^19^, therefore it could be considered as a metabolic disease. This theory was overtaken by the rapid developments in cancer genetic and protein biology. However, recent developments in cancerous signaling pathways suggest the participation of certain metabolic pathways in cancer cell proliferation^20^,^21^. This stimulated the scientific community to reinvestigate this aspect, and metabolism is now been included as one of the hallmarks of cancer. In the current study, ^1^H NMR-based metabonomics approach was employed to distinguish the metabonomic differences among acute lymphoblastic leukemia, acute myeloid leukemia, aplastic anemia patients, and healthy controls. The metabolic differences were highlighted in multiple metabolic pathways involving energy metabolism, protein biosynthesis, and metabolisms of fatty acids and choline, which probably involved biosynthesis of cell membranes. Differential metabolite levels in sera of patients against the healthy controls clearly indicated a shift of energy metabolism under the condition of acute leukemia.

Major amount of blood glucose is used in glycolysis for the fulfillment of energy requirements of cancer cells. When enough glucose is not produced from glycogen breakdown, a secondary metabolic pathway for glucose generation is adopted which consumes glucogenic amino acids, pyruvate, glycerol, and lactate. Higher blood glucose levels in ALL patients suggested that ALL patients had elevated secondary metabolic processes for constant supply of glucose as compared to AML and APA. The higher levels of lactate and low levels of other glucogenic amino acids including alanine, glutamine, histidine, lysine, valine, and proline in ALL patients’ sera further supported this inference. These findings suggest that at initial stages of disease, the TCA cycle was not activated by amino acids. However, consumption of amino acids for energy production is much faster in ALL than in AML.

Significantly lower levels of HDL and some unsaturated fatty acids were found in the blood of AML patients, as compared to controls. This is in accordance with previous findings^12^ and indicates that the variations of fatty acid metabolisms are associated with AML. This situation, along with the HDL depletion in blood, may be related to the increasing demands for lipids and cholesterols for tumor cell proliferation. Interestingly, in the case of ALL, unsaturated fatty acids were present in elevated levels. As it is proven that fatty acids can also serve as energy source, hence their elevated level in ALL may work as energy reservoir in cells and blood.

Choline plays important roles in phospholipid metabolism related to cell membranes.An elevation of phosphocholine (PC) along with total choline containing metabolites (tCho) has been reported in numerous *in vitro* and *in vivo* NMR studies of tumor cells^22^,^23^. This is now considered as a characteristic feature for aberrant choline phospholipid metabolisms in cancers. Lower levels for choline observed in the patients’ serum in this study probably due to the excessive need for choline and its derivatives during leukemic cell proliferation.

Long-term survivors of aplastic anemia have an increased risk of developing AML after immunosuppressive therapy, hence in order to relate their initial metabolome profile APA group was compared to AML. Interestingly, all the metabolites in Fig. 6 showed similar pattern and relative concentration of metabolites in AML was lowered in comparison to APA. Although the patients of AML has no past history of APA or any other malignancy, their similar initial metabolite profiling may conclude that APA and AML share the same metabolic pathways aberration in the course of disease.

In general, the observed trend of alterations in levels of glucose, phenylalanine, lactate, citrate, alanine, lysine, leucine, isoleucine, tyrosine, and valine was in agreement with the observations in oral cancer^24^. Similarly, serum choline concentration is increased in oral cancer. However, most of the discriminating metabolites observed in the sera of ALL and AML patients was not observed in the case of human hepatocellular carcinoma (HCC)^25^. Such opposite results are not unexpected as it is obvious that the different cancer cells showed different metabolic behavior and the nature of leukemia is very different from the solid tumors. However, our observations appear to be more consistent with the observations in oral cancer, and therapy-related myelodyspasia/acute myeloid leukemia (t-MDS/AML)^26^ than those in chronic lymphocytic leukemia (CLL)^27^. In a study using AML serum samples^12^, all the common identified metabolites showed the same pattern of down-regulation except glucose, which is up-regulated in the study mentioned. The differences in regulation of metabolites may be because of the intrinsically heterogeneous nature of the hematological malignances, the differences in disease stages and the clinical features of the controls.

## Conclusion

This study suggested that ALL and AML serum metabolic profiles are different from each other. They are also different as compared to healthy controls. Based on these observations, NMR-based metabonomics approach can be suitable for this profiling. The metabolic pathways involved in the separation of these samples are glycolysis, TCA cycle, lipoprotein changes, and choline and fatty acid metabolisms. It was noticed that larger cohort studies are necessary in future studies involving a combined approach of genomics, epigenomics, proteomics, metabonomics, and transcriptomics. It will also be interesting to investigate the probable clinical application of the NMR-based metabonomics in the early diagnosis of acute leukemia along with other hematological disorders. However, the current results firmly signify the potential of this approach for rapid and noninvasive diagnosis of acute leukemia in clinical studies.

## Additional Information

**How to cite this article**: Musharraf, S. G. *et al*. Serum metabonomics of acute leukemia using nuclear magnetic resonance spectroscopy. *Sci. Rep.* **6**, 30693; doi: 10.1038/srep30693 (2016).

## Supplementary Material

Supplementary Information

## Figures and Tables

**Table 1:** Average prediction results obtained by a default method of 7-fold internal cross validation of the software of PLS-DA and OPLS-DA models based on standard 1D, CPMG and diffusion edited spectra of serum from ALL, AML, APA and healthy control.

	R2	Q2	Sensitivity	Specificity	Classification rate
PLS-DA model					
Standard 1D	0.254	0.11	77.08	100	82.81
CPMG	0.291	0.086	79.17	100	84.38
Diffusion edited	0.234	0.099	66.67	32	75
OPLS-DA model					
Standard 1D	0.344	0.306	64.58	93.75	71.88
CPMG	0.62	0.492	87.5	100	90.63
Diffusion edited	0.416	0.308	67.71	100	75.78
