# Supplementary material for: Serum metabonomics of acute leukemia using nuclear magnetic resonance spectroscopy
Source: Sci Rep. 2016 Aug 2;6:30693. doi: 10.1038/srep30693 (PMC4969755; doi:10.1038/srep30693)
Supplement: Supplementary Information [file srep30693-s1.pdf]

Serum metabonomics of acute leukemia using nuclear magnetic resonance spectroscopy

*Syed Ghulam Musharraf,[1,2] Amna Jabbar Siddiqui,[2] Tahir Shamsi,[1,3] M. Iqbal Choudhary,[1,2,4] and Atta-ur-Rahman[1,2]

[1]Dr. Panjwani Center for Molecular Medicine and Drug Research, International Center for Chemical and Biological Sciences, University of Karachi, Karachi-75270, Pakistan;
[2]H.E.J. Research Institute of Chemistry, International Center for Chemical and Biological Sciences, University of Karachi, Karachi-75270, Pakistan;
[3]National Institute of Blood Diseases & Bone Marrow Transplantation, Karachi-75300, Pakistan.
[4]Department of Chemistry, College of Science, King Saud University, Riyadh-1145, Saudi Arabia.

*Corresponding author. Tel.: +92 021-34824924; 4819010; fax: +92 021-34819018-9.

E-mail address: musharraf1977@yahoo.com

Supplementary Information

Supplementary table S1: Clinical classification of patients.

	Subtypes	Number of patients
**AML**	AML without maturation	11
	AML with maturation	11
	Acute promyelocytic leukemia	3
	Acute myelomonocytic leukemia	2
	Acute monoblastic leukemia	1
	Not classified at the time of sampling	4
**ALL**	B-cell	2
	CALLA positive	5
	MPO negative	6
	Precursor B cell	5
	T cell	9
	Not classified at the time of sampling	5

CALLA: common acute lymphoblastic leukemia-associated antigen

MPO: Myeloperoxidase enzyme

<u>Subject characteristics</u>

Groups	Healthy control	ALL	AML	APA
Age in years				
Min–Max (median)	8-73(26)	2–61 (22.5)	5-75 (30)	7–55 (22.5)
Sex				
Male	16	23	19	23
Female	16	9	13	9

Supplementary Table S2: Cross validated-ANOVA table for assessing the reliability of the generated models

**PLS-DA model**						
	SS	DF	MS	F	p	SD
Total corr.	363	363	1			1
Regression	97.8659	36	2.7185	3.35282	$4.21x10^{-09}$	1.64879
Residual	265.134	327	0.810808			0.900449
**OPLS-DA model**						
	SS	DF	MS	F	p	SD
Total corr.	381	381	1			1
Regression	155.369	60	2.58948	3.68399	$2.74\ x10^{-14}$	1.60919
Residual	225.631	321	0.702901			0.838392

SS= sum of squares, DF= degree of freedom, MS= mean squares, F= F-test calculate value, p= p-value of the test, SD= standard deviation

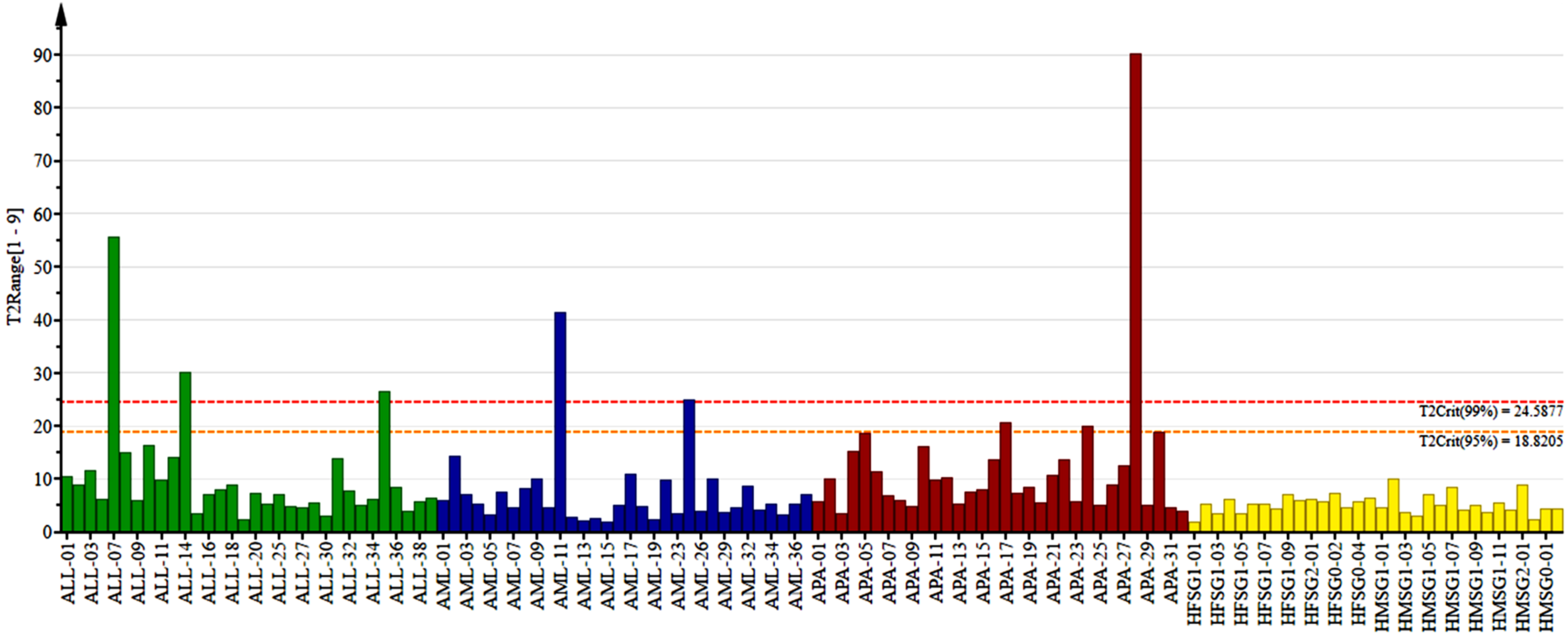

Supplementary Figure S1: Hotelling's T_2 plot with 99% and 95% confidence limits showing outliers.

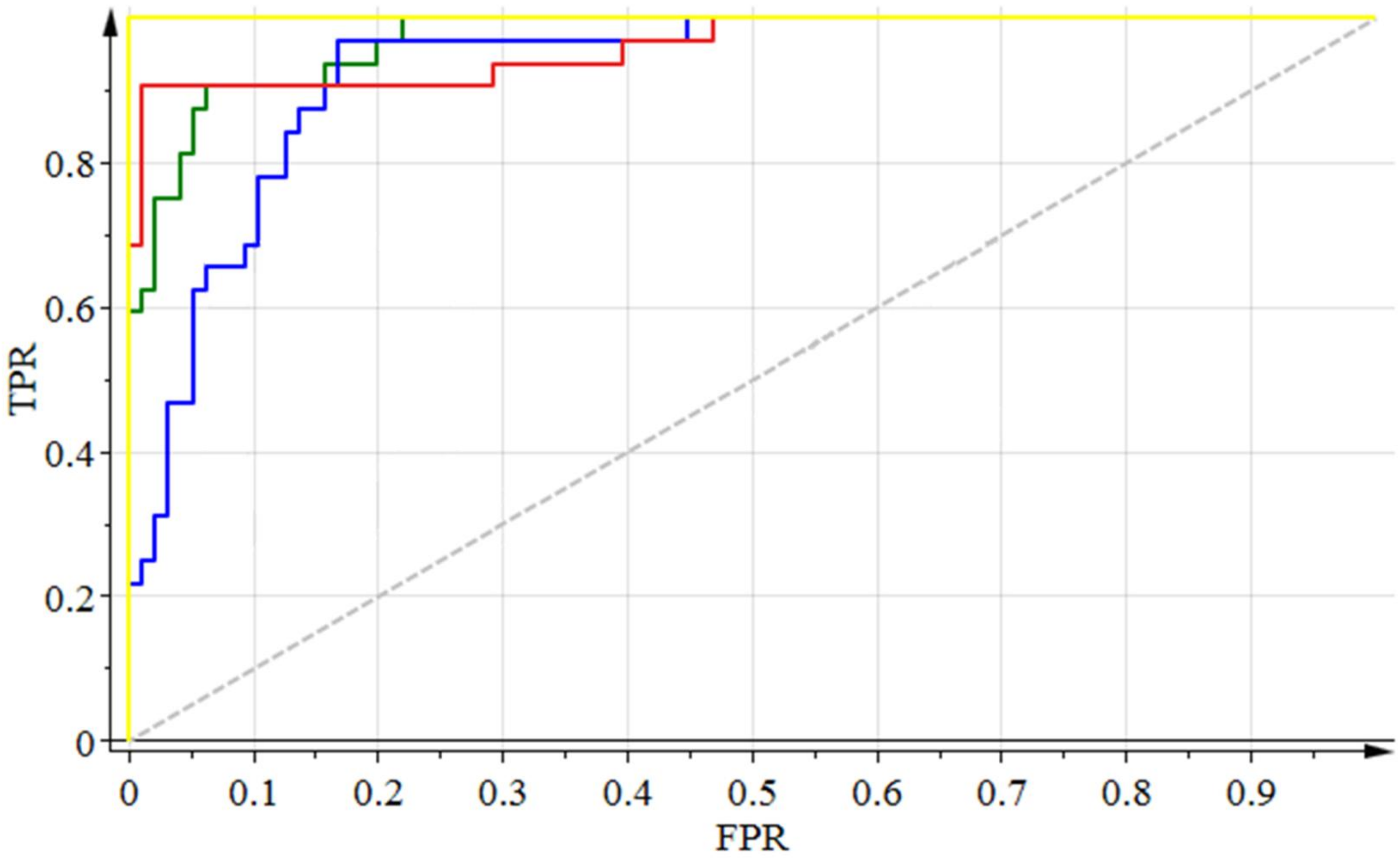

Supplementary Figure S2: Reciever operating characteristic (ROC) plot for OPLS-DA model showing sensitivity on y-axis and 1-specificity on x-axis [ALL (green), AML (blue), APA (red) and healthy control (yellow)].
